# Mechanism of Ca^2+^-Dependent Pro-Apoptotic Action of Selenium Nanoparticles, Mediated by Activation of Cx43 Hemichannels

**DOI:** 10.3390/biology10080743

**Published:** 2021-08-03

**Authors:** Egor A. Turovsky, Elena G. Varlamova

**Affiliations:** Federal Research Center “Pushchino Scientific Center for Biological Research of the Russian Academy of Scienes”, Institute of Cell Biophysics of the Russian Academy of Sciences, 142290 Pushchino, Russia

**Keywords:** selenium nanoparticles, cancer, apoptosis, calcium signaling, connexins, purinoreceptors, gene expression

## Abstract

**Simple Summary:**

The development of new methods of anticancer therapy is an urgent task of modern biology. The use of selenium and its compounds as anticancer drugs have already been shown to be effective. At the same time, the problem of selective delivery of selenium to organs and tissues remains. Nanoparticles are a promising and effective form of selenium delivery. This work is devoted to the study of the molecular and cellular mechanisms of action of selenium nanoparticles on glioblastoma cells. Using fluorescence microscopy and PCR, we were able to show for the first time the complete Ca^2+^ signaling pathway activated by selenium nanoparticles and the correlation between an increase in Ca^2+^ ions in the cytosol of cells with the induction of apoptosis. The presented study contributes to understanding the fundamental mechanisms of activation of programmed cell death in cancer tissue.

**Abstract:**

To date, there are practically no data on the mechanisms of the selenium nanoparticles action on calcium homeostasis, intracellular signaling in cancer cells, and on the relationship of signaling pathways activated by an increase in Ca^2+^ in the cytosol with the induction of apoptosis, which is of great importance. The study of these mechanisms is important for understanding the cytotoxic effect of selenium nanoparticles and the role of this microelement in the regulation of carcinogenesis. The work is devoted to the study of the role of selenium nanoparticles obtained by laser ablation in the activation of the calcium signaling system and the induction of apoptosis in human glioblastoma cells (A-172 cell line). In this work, it was shown for the first time that the generation of Ca^2+^ signals in A-172 cells occurs in response to the application of various concentrations of selenium nanoparticles. The intracellular mechanism responsible for the generation of these Ca^2+^ signals has also been established. It was found that nanoparticles promote the mobilization of Ca^2+^ ions from the endoplasmic reticulum through the IP_3_-receptor. This leads to the activation of vesicular release of ATP through connexin hemichannels (Cx43) and paracrine cell activation through purinergic receptors (mainly P2Y). In addition, it was shown that the activation of this signaling pathway is accompanied by an increase in the expression of pro-apoptotic genes and the induction of apoptosis. For the first time, the role of Cx43 in the regulation of apoptosis caused by selenium nanoparticles in glioblastoma cells has been shown. It was found that inhibition of Cx43 leads to a significant suppression of the induction of apoptosis in these cells after 24 h treatment of cells with selenium nanoparticles at a concentration of 5 µg/mL.

## 1. Introduction

Apoptosis is an important process of selective destruction of damaged or defective cells in the process of vital activity and a violation of this process can lead to the development of cancer [1]. There are three main pathways for the induction of apoptosis, the mitochondrial pathway, the death receptor pathway, and the endoplasmic reticulum (ER) pathway, which ultimately lead to the activation of caspase-3 and proteolysis of cellular components [2]. In the case of induction of apoptosis via the ER-signaling pathway, activation of caspase-3 occurs as a result of disruption of ER-homeostasis, depletion of Ca^2+^ stores through RyR and IP_3_R, and activation of caspase-12, identified in rodents, while in humans its role is presumably played by caspase-4 [3,4].

Ca^2+^ ions as a secondary messenger regulate the action of more than 100 hormones and neurotransmitters. Numerous cellular functions, determining both the normal physiology of cells and the processes of their death, including through apoptosis, activating both the early “commitment phase” through IP_3_R and the later “execution phase” [5,6,7,8]. One of the mechanisms responsible for the induction of apoptosis is the transport of proapoptotic factors from cell to cell through the gap junctions formed by connexins. Vertebrate connexins (Cx) are represented by 20 protein isoforms containing four transmembrane domains and differing from each other in the length of the cytoplasmic domain. Connexins form channels between neighboring cells and are responsible for the transport of ions and small molecules [9,10]. Molecules that induce apoptosis through gap junctional communication are Ca^2+^, IP_3_, and cAMP ions, with the ultimate target of caspases and the opening of mitochondrial permeability transition pores [11].

Unlike other connexins, Cx43 has the ability to form intercellular channels and hemichannels that open to the external environment and thereby regulate cellular metabolism [12,13]. It is known that Cx43, together with aquaporin-4, are involved in the formation of glioma-induced brain edema [14]. It is known that the expression of Cx43 in human glioblastoma cells increases their sensitivity to chemotherapy. Thus, patients with glioblastoma with a reduced level of Cx43 expression have poor prognosis compared to those who present with histologically and clinically similar disease but whose neoplasm expresses high levels of Cx43 and low levels of bcl-2 [15]. The search for targeted anticancer therapy has been an urgent task in biology for many years. There is a wide range of biological molecules with pro and antioxidant properties, among which selenium compounds play an important role. Selenium (Se) is present as selenocysteine in 25 human proteins and enzymes and plays a protective role in several disease conditions, e.g., hypercholesterolemia, some cancers, and cardiovascular disorders [16]. Se can act as a pro and antioxidant depending on concentration and disease. At non-lethal doses, Se usually induces apoptosis and suppresses the proliferation of cancer cells by inducing oxidative stress [17]. To reduce toxic effects and stabilize Se, recently, technologies for creating nanoparticles based on this microelement have often been used [18,19]. In medicine, the use of SeNP can solve one of the main problems—the resistance of cancer cells to chemotherapeutic drugs. SeNP are also more selective and effective in damaging tumor cells [20,21,22]. It was shown that the cytotoxic effect of SeNP is accompanied by the activation of various signaling pathways of apoptosis [23]. However, the focus of this work was the study of the Ca^2+^-dependent anticancer effect of selenium nanoparticles obtained by laser ablation in a liquid.

## 2. Materials and Methods

### 2.1. Reagents

From Sigma-Aldrich (St. Louis, MO, USA): Adenosine 5′-triphosphate disodium salt hydrate (ATP, A1852), DMEM (D6429), Hanks′ Balanced Salt Solution (HBSS, H4641), HEPES sodium salt (H7006), Monensin (M5273), Nystatin (N6261), Amiloride (PHR1839), Xestospongin C (X2628), MRS-2179 (M3808), U73122 (U6756), Thapsigargin (T9033), Ryanodine (559276), Carbenoxolone (C4790), ^10^Panx1 (SML2152), Bafilomycin A1 (19-148), Apyrase (A6237), Gap26 (SML3074). From Molecular probes (Eugene, OR, USA): Hoechst 33,342 (H1399), Propidium iodide (P1304MP). From Evrogene (Moscow, Russia): MMLV reverse transcriptase (SK022S), SYBR Green I PCR Master Mix (PK147L); Triethylammonium salt (TNP-ATP, Ann Arbor, MI, USA, 20902). From Thermo Fisher Scientific (Waltham, MA, USA): Fura-2AM (Cat. #F1221), Fetal Bovine Serum (10099141). Selenium nanoparticles (kindly provided by Dr. S.V. Gudkov, Prokhorov General Physics Institute of the Russian Academy of Sciences, Moscow, Russia).

### 2.2. Cell Culture

**Figure 1:**
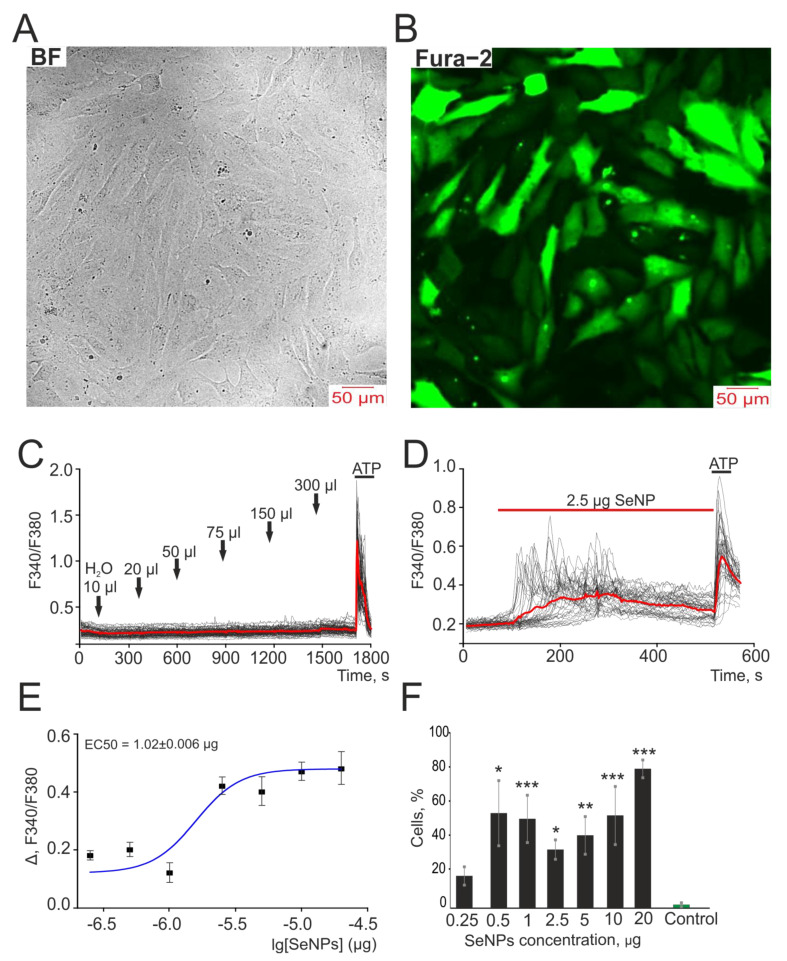
Application of SeNP dose-dependently induces the generation of Ca^2+^ signals in human A-172 glioblastoma cells. At the end of the experiment, 10 μM adenosine triphosphate (ATP) was added. (**A**,**B**) Images of A-172 cell culture in bright field microscopy (**A**) and Fura-2 fluorescence (**B**) at an excitation of 380 nm. (**C**) The absence of Ca^2+^ signals when different volumes of distilled water are applied to the cells. (**D**) Generation of Ca^2+^ signals in 36 ± 7% of cells in response to application of 2.5 µg/mL SeNP. (**E**) Dependence of the amplitude of Ca^2+^ responses of cells on the growth of SeNP concentration and its approximation by a sigmoid function. (**F**) Percentage of cells in culture that respond to the application of 300 µL distillated water (control) and various concentrations of SeNP. Ca^2+^ signals in cells and their average value (red curve) are shown. The differences between the 0.25 µg/mL SeNP and other concentrations of SeNP are marked with asterisks—* *p* < 0.05, ** *p* < 0.01, and *** *p* < 0.001. n/s—differences insignificant.

Cell line A-172 were purchased from ATCC (Manassas, VA, USA) and were grown on round coverslips for 48 h in a CO_2_-incubator in DMEM medium supplemented with 10% fetal bovine serum until a confluence of 80–95% (Figure 1A,B) was achieved. Cell cultures were used from the third passage. We checked our continuous cell culture for mycoplasma contamination every three months by PCR as recommended by Drexler and Uphoff [24]. No mycoplasma contamination detected.

### 2.3. Assessment of Cell Viability and Apoptosis

Cell death (apoptosis or necrosis processes) in the cell culture was assessed by simultaneous staining of cells with Propidium iodide (PI, 1 µM) and Hoechst 33,342 (HO342, 1 µM). Viable cells are not permeable to PI, while Hoechst 33,342 penetrates through the plasma membrane, staining the chromatin. According to a commonly used method [25,26], A-172 cells were defined as apoptotic if the intensity of Hoechst 33,342 fluorescence was 3–4 times higher compared to Hoechst 33,342 fluorescence in healthy cells, indicating chromatin condensation which can occur as a result of apoptosis induction. The fluorescence of the probes was registered with a fluorescent system based on an inverted fluorescent microscope, Axio Observer Z1, equipped with a high-speed monochrome CCD-camera Hamamatsu ORCA-Flash 2.8. The Lambda DG-4 Plus illuminator (Sutter Instruments, Novato, CA, USA) was used as a source of excitation. To excite and record fluorescence of the probes we used: Filter Set 01 with excitation filter BP 365/12, beam splitter FT395, emission filter LP 397; Filter Set 20 with excitation filter BP 546/12, beam splitter FT560, emission filter BP 575–640. We used objective HCX PL APO 20.0 × 0.70 IMM UV, refraction index 1.52. Camera settings were 500 pixels × 500 pixels (Voxel Size 0.724 µm × 0.723 µm), binning 2 × 2, resolution 14 bits. Five different fields of view were analyzed for each coverslip with cells. Each experiment was repeated three times with separate cell cultures. To simultaneously monitor apoptotic and healthy cells after SeNP treatment with fluorescence microscope, Apoptosis/Necrosis Detection Kit was used. Incubation of cells was carried out for 30 min with 2.5 and 5 µg/mL concentrations of SeNP, which we used for calcium imaging experiments. Then, cells were washed 1–2 times and resuspended with assay buffer. To detect apoptotic cells, Apopxin Green Indicator was used. Apoptotic cells were visualized using the FITC channel (Ex/Em = 490/525 nm). For staining necrotic cells, we used 7-aminoactinomycin D (Ex/Em = 550/650 nm). To detect healthy cells, CytoCalcein 450 was used and cells were visualized using the violet channel (Ex/Em = 405/450 nm).

### 2.4. Fluorescent Ca^2+^ Measurements

Experiments were carried out in the daytime. The measurements of [Ca^2+^]_i_ were performed by fluorescence microscopy using Fura-2/AM. A-172 cells were loaded with the probe dissolved in Hanks Balanced Salt Solution (HBSS) composed of (mM): 156 NaCl, 3 KCl, 2MgSO_4_, 1.25 KH_2_PO_4_, 2CaCl_2_, 10 glucose, and 10 HEPES, pH 7.4 at 37 °C for 40 min with subsequent 15 min washout. To measure free cytosolic Ca^2+^ concentration, we used the Carl Zeiss Cell Observer and an inverted motorized microscope Axiovert 200M with a high-speed monochrome CCD-camera AxioCam HSm with a high- speed light filter replacing system, Ludl MAC5000. Fura-2 excitation and registration were recorded using a 21HE filter set (Carl Zeiss, Germany) with excitation filters BP340/30 and BP387/15, beam splitter FT-409, and emission filter BP510/90, objective lens HC PL Fluotar 10×/0.3 Dry, refraction index 1, excitation light source HBO 103W/2. Camera settings were 500 pixels × 500 pixels (Voxel Size 0.724 µm × 0.723 µm), binning 2 × 2, resolution 14 bits. Background fluorescence was subtracted frame by frame using the Math Subtract plugin in ImageJ. Calcium responses were shown as a ratio of Fura-2 fluorescence intensities. Therefore, we determined the amplitudes of Ca^2+^ responses to SeNPs as (Δ)—Fmax–Fmin of Fura-2 fluorescence and an increase in Fura-2 fluorescence reflects a linear [Ca^2+^]_i_ increase in response to receptor agonists. ImageJ 2002 software (RRID: SCR_003070) was used to analyze data.

### 2.5. Extraction of RNA and Real-Time Polymerase Chain Reaction (RT-qPCR)

Total RNA from A-172 cells after 24 h of treatment with various concentrations of SeNP was isolated using ExtractRNA reagent according to the manufacturer’s instructions (Evrogene, Russia). The concentration and purity of the total RNA were determined spectrophotometrically at 260/280 nm. First-strand cDNA was synthesized from 1–2 µg of total RNA using MMLV reverse transcriptase according to the manufacturer’s instructions (Evrogene, Russia). Real-time qPCRs were performed in a 25 µL reaction mixture containing SYBR Green I PCR Master Mix (Evrogene, Russia) and 300 nM of the appropriate primers (Table 1). The PCR procedure consisted of 94 °C for 2 min followed by 35 cycles of 94 °C for 1 min, 60 °C for 30 s, and 72 °C for 30 s. Glyceraldehyde-3-phosphate dehydrogenase (GAPDH) was used as an internal control for normalization and results were expressed as 2–∆(∆Ct).

### 2.6. Preparation of Selenium Nanoparticles

Selenium nanoparticles were obtained by laser ablation in deionized water. The solid target was placed at the bottom of a cuvette under a thin layer of water. In this state, the solid target was irradiated with a laser beam (λ = 1064 nm; T = 4–200 ns; f = 20 kHz; P = 20 W; E_p_ = 1 mJ). The laser beam was mixed on the target using a galvanomechanical scanner TM 2D (Ateko, Moscow, Russia). Depending on the characteristics of the laser radiation, the speed and trajectory of the laser beam, it is possible to obtain colloidal solutions of selenium nanoparticles with specified geometric parameters. The nanoparticle size was characterized using a DC24000 analytical centrifuge (CPS Instruments, Prairieville, LA, USA). The nanoparticle concentration and hydrodynamic radius were evaluated using the Zetasizer Ultra Red Label (Malvern, UK). The morphology of the nanoparticles was studied by electron energy loss spectroscopy using a 200FE transmission electron microscope (Carl Zeiss, Berlin, Germany).

The evolution of nanoparticle size distribution was investigated using a disk analytical centrifuge. It was found that the obtained preparation of selenium nanoparticles has a monomodal size distribution. The average nanoparticle size is about 0.1 µm, the half-width is in the range 0.075–0.125 µm.

### 2.7. Statistical Analysis

All presented data were obtained from at least three cell cultures from 2–3 different passages. All values are given as mean ± standard error (SEM) or typical Ca^2^+ signals. Statistical analyses were performed by paired t-test. ImageJ, Origin 2016 (OriginLab, Northampton, MA, USA), and Prism GraphPad 7 (GraphPad Software, RRID: SCR_002798) software was used for data and statistical analysis.

## 3. Results

### 3.1. SeNP Dose-Dependently Induce the Generation of Ca^2+^ Signals in A-172 Cells

In order to neutralize the contribution of the solvent of nanoparticles (distilled water), we preliminarily carried out experiments in which we monitored the generation of calcium signals in A-172 cells in response to the addition of various volumes of water. According to the results presented in Figure 1C, the addition of a solvent to the cells did not lead to the generation of spontaneous Ca^2+^ signals, while the application of already 2.5 μg/mL SeNP led to Ca^2+^ signals in 33 ± 8% of cells with average amplitude of 0.25, and most cells responded with Ca^2+^ oscillations (Figure 1D). It was shown that with an increase in SeNP concentration, an increase in the amplitude of Ca^2+^ signals and the number of cells generating these signals was observed, and the EC50 value was 1.02 ± 0.006 µg. Therefore, for further experiments, we decided to use a double concentration—2.5 µg (Figure 1E,F).

### 3.2. SeNP Enters A-172 Cells by Activating Micropinocytosis and Clathrin-Associated Endocytosis

It is well known that the main mechanism for the entry of nanodrugs and therapeutic genes into the cell is endocytosis which determines the targeted delivery of nanoparticles [27]. Moreover, there are several types of endocytosis: micropinocytosis, caveolar, and clathrin-associated endocytosis. In order to understand by what endocytosis SeNP enter A-172 cancer cells, a series of experiments was carried out using blockers of each type of endocytosis.

**Figure 2:**
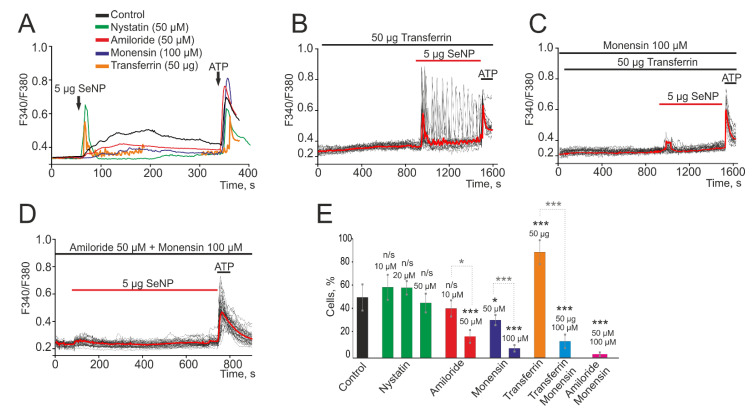
Contribution of micropinocytosis, caveolar, and clathrin-associated endocytosis to the generation of Ca^2+^ signals in A-172 cells upon application of 5 µg/mL SeNP. (**A**) Average values of Ca^2+^ signals caused by the application of 5 µg/mL SeNP over several tens of cells in the control (black curve) and after 2 h incubation of cells with a blocker of caveolar endocytosis (green curve, Nystatin, 50 µM), micropinocytosis (red curve, Amiloride, 50 µM), and clathrin-associated endocytosis (purple curve, Monensin, 100 µM). The potentiating effect of a receptor-mediated clathrin-associated endocytosis activator, Transferrin (orange curve, 50 µM), on Ca^2+^ signals upon SeNP application. (**B**) Application of SeNP after incubation with Transferrin tends to increase the amplitude of Ca^2+^ signals. (**C**) The blockade of clathrin-associated endocytosis with Monensin reverses the potentiating effect of Transferrin on Ca^2+^ signals upon application of SeNP. (**D**) Complete suppression of Ca^2+^ signals in cells in response to the application of 5 µg/mL SeNPs after simultaneous inhibition of micropinocytosis and clathrin-associated endocytosis. At the end of the experiment, 10 μM adenosine triphosphate (ATP) was added. (**E**) The number of cells in percentage ratio, reacting with Ca^2+^ signals upon application of 5 µg/mL SeNPs during inhibition of various types of endocytosis or activation of a receptor-mediated clathrin-associated endocytosis. The differences between the control and experimental groups are marked with asterisks—* *p* < 0.05 and *** *p* < 0.001. n/s—differences insignificant.

It was found that incubation of cells for 2 h with a blocker of caveolar endocytosis (Nystatin) and subsequent application of 5 µg/mL SeNPs contributed to a slight increase in the amplitude of the Ca^2+^ signal to 0.32 compared to 0.27 in the control (Figure 2A). While the use of a micropinocytosis blocker (Amiloride) at a concentration of 50 μM led to a decrease in Ca^2+^ signals to 0.1 (suppression by 63% relative to the control) and a decrease in the percentage of responding cells to 16 ± 10% (Figure 2A,E), with lower concentrations amiloride, Amiloride proved to be ineffective. A similar effect was observed after treatment of cells with an inhibitor of clathrin-associated endocytosis (Monensin) at a concentration of 100 μM during 2 h. There was a decrease in the amplitude of Ca^2+^ signals to 0.07 (a decrease by 75% compared to the control) (Figure 2A, purple curve) and the number of responders cells up to 11 ± 5 (Figure 2E). Lower concentrations of this blocker (50 µM) were found to be less effective (Figure 2E). Application of a receptor-mediated clathrin-associated endocytosis activator, Transferrin, does not induce Ca^2+^ signals in A-172 cells. However, there is a trend towards an increase in the amplitude of Ca^2+^ signals for the application of SeNP (Figure 2B) and an increase in the number of responding cells to almost 100% (Figure 2E). The potentiating effect of Transferrin was abolished after 2 h of cell incubation with Monensin and Ca^2+^ responses were observed, probably induced by the micropinocytosis mechanism (Figure 2C,E). A significant effect in suppressing the amplitude of Ca^2+^ signals and the number of cells generating these signals in response to the application of 5 µg/mL SeNPs during 2 h was observed with the combined use of Amiloride and Monensin blockers (Figure 2D,E). In this case, no cytotoxic effects were revealed since we have not recorded a global increase in [Ca^2+^]_i_ and cells respond to the application of ATP at the end of the experiments. Besides, after incubation of cell cultures with 2.5 and 5 µg/mL SeNPs for 30 min using the Apoptosis/Necrosis Detection Kit assay, no cells with apoptosis and necrosis were detected (Supplementary, Figure S1). Thus, for the entry of SeNP into A-172 cells, most likely, two types of endocytosis are activated: micropinocytosis and clathrin-associated endocytosis.

### 3.3. SeNP Induce Ca^2+^ Signals through the Activation of the Phosphoinositide Signaling Cascade and the Mobilization of Ca^2+^ Ions from the Thapsigargin-Sensitive ER Pool

**Figure 3:**
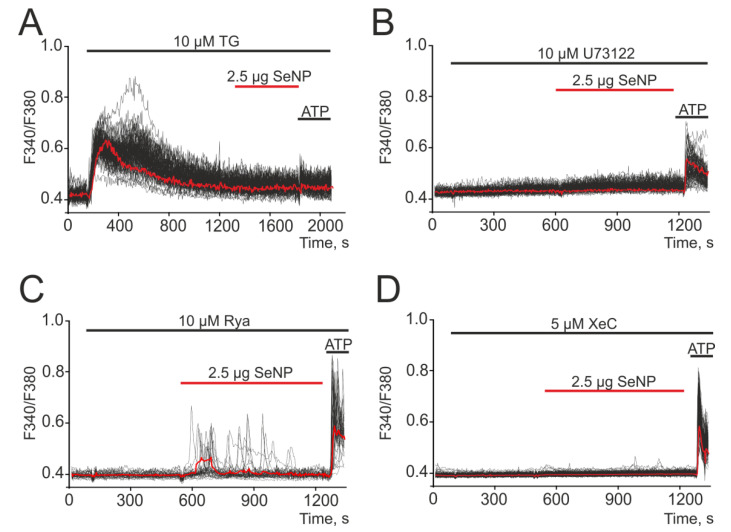
Generation of Ca^2+^ signals in A-172 cells in response to the application of 2.5 μg/mL SeNP after depletion of the ER with Thapsigargin (**A**, TG, 10 μM), in the presence of 10 μM (**B**) of PLC inhibitor—U73122, antagonists of ryanodine (**C**, ryanodine, Rya, 10 µM) and IP3 receptors (**D**, Xestospongin C, XeC, 5 µM). At the end of the experiment, 10 μM adenosine triphosphate (ATP) was added. Here, Ca^2+^ signals of cells in one experiment and their and their average value (red curve) are presented.

Electro-nonexcitable cells use intracellular depots to generate Ca^2+^ signals [28], most often the ER depot. The mechanisms of Ca^2+^ signals, including oscillatory modes, can be divided into two large classes depending on the use of IP_3_R or RyR receptors for the mobilization of Ca^2+^ from intracellular compartments [29]. To distinguish the possible pathways for Ca^2+^ release, we used PLC, IP_3_R, and RyR inhibitors. After the depletion of the ER calcium pool using Thapsigargin, a complete suppression of Ca^2+^ responses to the application of SeNP and ATP was observed (Figure 3A). Mobilization of Ca^2+^ ions from the Thapsigargin-sensitive ER pool can occur via IP_3_R or RyR receptors, and the activation of the former requires PLC activation. Inhibition of PLC with U73122 resulted in complete suppression of Ca^2+^ responses of all cells to the addition of SeNP (Figure 3B). Inhibition of A-172 cell signals to SeNP application also occurred after incubation with Xestospongin C (Figure 3D, XeC), an IP_3_R antagonist, but Ca^2+^ signals were retained against the background of the RyR blocker (ryanodine) (Figure 3C, Rya).

### 3.4. SeNP Involve ATP-Release Mechanisms through the Activation of Connexin Hemichannels in the A-172 Cell Line

**Figure 4:**
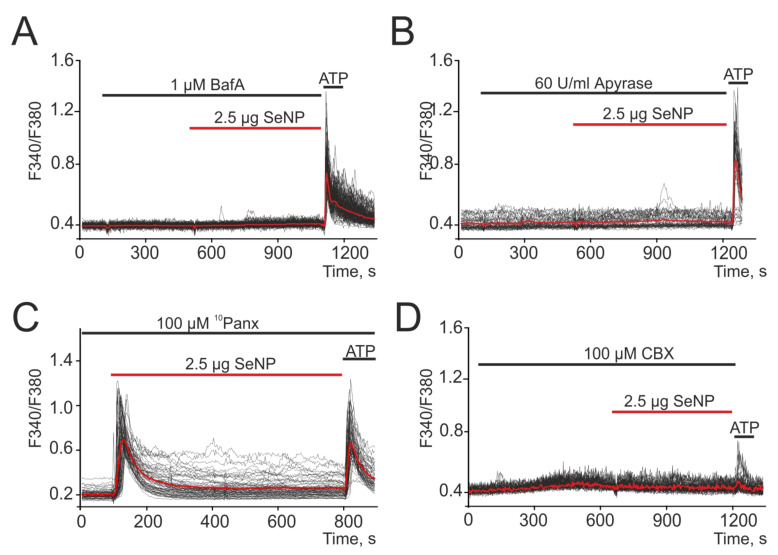
Suppression of Ca^2+^ signals in A-172 cells in response to the application of 2.5 µg SeNPs in the presence of a vesicular secretion blocker Bafilomycin A1 (**A**, Baf A1, 1 µM), apyrase (**B**, 60 U/mL), an enzyme that breaks down ATP and connexin blocker Carbenoxolone (**D**, CBX, 100 µM). (**C**) Pannexin blocker, ^10^Panx (100 µM), did not affect the Ca^2+^ signals of cells in response to SeNP. At the end of the experiment, 10 μM adenosine triphosphate (ATP) was added. Here, Ca^2+^ signals of cells in one experiment and their average value (red curve) are presented.

It is known that secretion is one of the key mechanisms of cellular communication. Therefore, a series of experiments was carried out to investigate the role of vesicular secretion in the generation of Ca^2+^ signals in response to the application of 2.5 µg/mL SeNP. When cells were exposed to the vesicular secretion blocker Bafilomycin A1 (Baf A1), a complete suppression of Ca^2+^ responses to the application of 2.5 µg/mL SeNP was observed, which indicates the participation of secretion in the mechanism of increasing [Ca^2+^]_i_ under the action of SeNP in A-172 cells (Figure 4A).

It is known that in astrocytes, which, much like glioblastoma, are also electrically excitable cells, an increase in [Ca^2+^]_i_ causes the release of glutamate and other transmitters, primarily ATP [7]. When studying the contribution of ATP secretion to the activation of Ca^2+^ responses, A-172 cells were pretreated with an ATP-degrading enzyme (Apyrase, 60 U/mL) and then 2.5 µg/mL SeNP were added. The results shown in Figure 4B indicate complete inhibition of Ca^2+^ responses mediated by the action of SeNP, which underlines the important role of ATP secretion in this regulation.

Intercellular contacts formed by connexins or pannexins can also be involved in the release of ATP and other active substances, both in neighboring cells and in the extracellular environment [30]. Therefore, we investigated their role in the generation of Ca^2+^ signals in response to the application of 2.5 µg/mL SeNP. It was found that the pannexin blocker (^10^Panx) did not affect the generation of Ca^2+^ signals by cells in response to the application of SeNP (Figure 4C), which excludes the contribution of intercellular channels formed by this protein. Using a connexin blocker (Carbenoxolone, CBX), complete suppression of Ca^2+^ signals in A-172 cells in response to application of SeNPs was shown (Figure 4D).

### 3.5. SeNP Generate Ca^2+^ Signals in A-172 Cells by Activating P2Y Purinoreceptors

As shown above, vesicular ATP secretion by SeNP triggers a complex signaling cascade. In order to understand which of the specific receptors is activated by ATP secretion in response to the application of SeNP in A-172 cells, a series of experiments was carried out using purinergic receptor antagonists.

**Figure 5:**
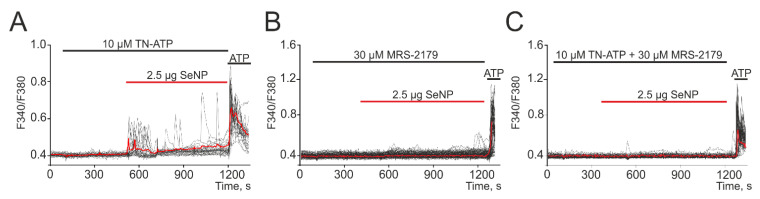
Generation of Ca^2+^ signals in A-172 cells in response to the application of 2.5 µg/mL SeNP in the presence of P2X receptors antagonist—TN-ATP (**A**, 10 µM), P2Y—receptors-MRS-2179 (**B**, 30 µM), and their combined action (**C**, 10 µM TN-ATP + 30 µM MRS-2179). At the end of the experiment, 10 μM adenosine triphosphate (ATP) was added. Here, Ca^2+^ signals of cells in one experiment and their average value (red curve) are presented.

It was found that treatment of cells with antagonist of P2X receptors (TN-ATP) led to only partial suppression of Ca^2+^ signals upon application of SeNP (Figure 5A) and a decrease in the number of cells generating these signals to 14 ± 6%. At the same time, the antagonist of P2Y receptors (MRS-2179) suppressed signals for application of 2.5 µg/mL SeNP in almost all cells (Figure 5B) and only in single cells were low-amplitude Ca^2+^ signals recorded. Incubation of cells together with antagonists of P2X- and P2Y-purinoreceptors contributed to the complete suppression of Ca^2+^ signals in all cells when exposed to SeNP (Figure 5C).

### 3.6. SeNPs Dose-Dependently Activate the Apoptosis Process in Human Glioblastoma Cells. Connexin-43 Contribution

**Figure 6:**
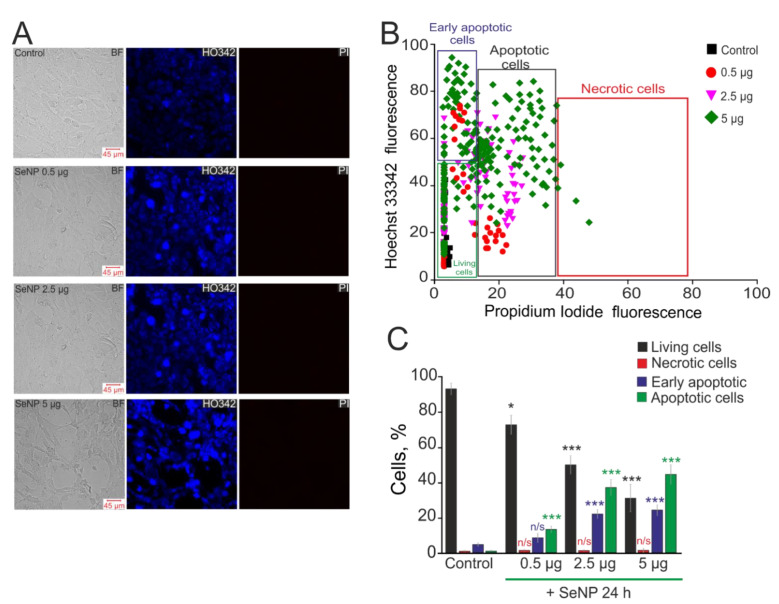
The effect of different concentrations of SeNP on induction of apoptosis and necrosis in the A-172 cells. (**A**) Double staining of cells with Hoechst 33,342 (HO342) and Propidium iodide (PI) after incubation with SeNP. (**B**) Cytogram of the viability of A-172 cells. *X*-axis—the intensity of PI fluorescence; *Y*-axis—the intensity of Hoechst 33,342 fluorescence. Cells were stained with the probes after 24 h of SeNP application. (**C**) The percentage of living cells (black column) and cells in which the processes of early apoptosis (violet column), apoptosis (green column), and necrosis (red column) were detected after incubation with SeNP. The differences between the control and experimental groups are marked with asterisks—* *p* < 0.05 and *** *p* < 0.001. n/s—differences insignificant.

It is known that selenium-containing compounds have shown high efficiency as anticancer drugs, and one of the key mechanisms in this case is the induction of apoptosis [31]. In this work, 24 h exposure of A-172 cells to various concentrations of SeNP (0.5 µg/mL, 2.5 µg/mL, and 5 µg/mL) led to the induction of both early and late stages of apoptosis, as evidenced by an increase in the fluorescence of the Hoechst 33,342 probe and the propidium iodide probe (Figure 6). An extremely low percentage of apoptotic cells were observed in intact cells (no more than 3 ± 2%, including 8 ± 3% of them at the early stages of apoptotic death). In addition, there was no necrotic death of A-172 cells in response to the application of SeNP (Figure 6A—PI; Figure 6B,C). After 24 h incubation of cells with 0.5 µg/mL SeNP, only a small number of them were in a state of apoptosis (16 ± 4% at the early stage and 19 ± 6% at the late stage) (Figure 6). An increase in SeNP concentrations resulted in a proportional increase in the number of apoptotic cells. Thus, when cells were treated with 2.5 µg/mL SeNP, we registered 27 ± 6% and 29 ± 4% of cells at the early and late stages of apoptosis, respectively, with the application of 5 µg/mL SeNP—41 ± 8 and 52 ± 11% of cells.

**Figure 7:**
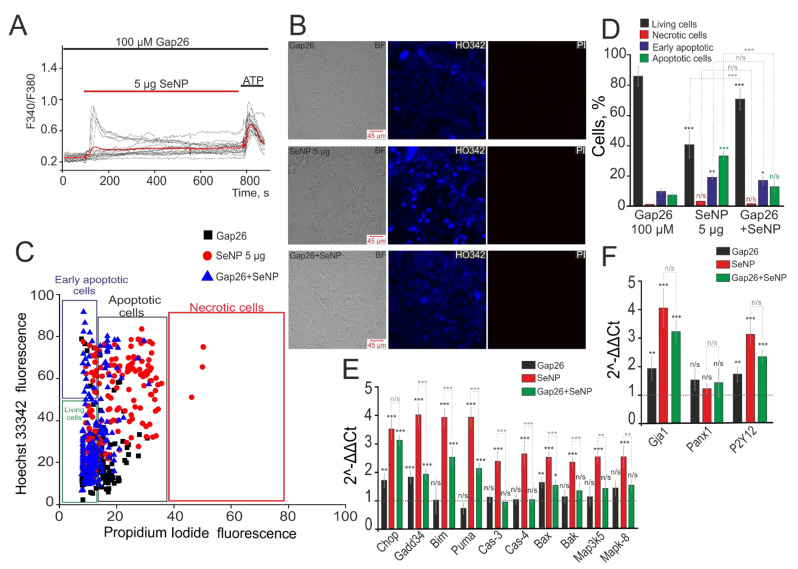
Inhibition of Cx43 suppresses the induction of apoptosis in A-172 cells induced by the application of 5 µg/mL SeNP. (**A**) Suppression of Ca^2+^ signals in 87 ± 11% of cells after incubation with 100 μM Gap26 for 30 min and subsequent incubation with 5 μg/mL SeNP for 24 h. (**B**) Identification of cells in the early and late stages of apoptosis or necrosis after incubating with 100 µM Gap26 and 5 μg/mL SeNP separately and together. (**C**) Cytogram demonstrating the viability of A-172 cells. *X*-axis—the intensity of PI fluorescence; *Y*-axis—the intensity of Hoechst 33,342 fluorescence. (**D**) The number of cells in the percentage ratio that are in the early (violet column) or late stages (green column) of apoptosis or necrosis (red column) and living cells (black column). (**E**,**F**) Real-time PCR results showing changes in mRNA expression of the main pro-apoptotic genes (**E**) and genes encoding receptors (**F**) in response to treatment of A-172 cells with 100 µM Gap26 (black columns), 5 μg/mL SeNP (red columns), and Gap26+SeNP (green columns). The horizontal line corresponds to the expression of mRNA of the studied genes in control samples (without some compounds application). The differences are marked with asterisks—* *p* < 0.05, ** *p* < 0.01, and *** *p* < 0.001. n/s—differences insignificant.

It is well known that apoptosis is closely related to calcium signaling and its induction is regulated by Ca^2+^ ions [5]. Since the central link in the generation of Ca^2+^ signals by A-172 cells in response to SeNP are connexin half channels, we assumed their participation in the process of apoptosis activation in these cells. We used connexin mimetic peptide (Gap26) to inhibit Cx43. It was shown that, in response to the application of 5 µg/mL SeNP, Gap26 suppressed Ca^2+^ signals in 87 ± 11% of cells after 30 min of exposure to the cells (Figure 7A). At the same time, incubation of cells with Gap26 (100 µM) did not affect the induction of apoptosis, but after the application of 5 µg/mL SeNP, a decrease in the density of the cell culture (Figure 7B), activation of the early stage (21 ± 7% of cells) and late stages (38 ± 9% of cells) of apoptosis were observed (Figure 7C,D). However, the application of 5 µg/mL SeNP against the background of Gap26 led to a decrease in cells with an early stage (19 ± 6%) and a late stage (14 ± 9%) of apoptosis (Figure 7C,D).

Induction of apoptosis requires a branched cascade of events involving changes in the expression of a number of genes encoding pro-apoptotic proteins. In addition, a series of experiments was carried out to investigate changes in the expression of a number of pro-apoptotic genes in A-172 cells in response to the application of 5 µg/mL SeNP during 24 h. The results obtained using the real-time PCR method indicate that incubation of A-172 cells with SeNP (Figure 7F, red bars) increased the expression of genes for pro–apoptotic proteins (CHOP, GADD34, BIM, PUMA, BAX, BAK) more than 2–4 times. Increased expression of pro-apoptotic proteins of the BCL-2 family (BAX and BAK) may indicate the activation of the mitochondrial apoptosis pathway since both proteins are known to increase the permeability of the outer mitochondrial membrane and disrupt its integrity. In addition, these pro-apoptotic proteins can be activated by other members of the BCL-2 family, for example, BIM and PUMA, the expression of which was increased more than 3–4 times in response to the application of 5µg/mL SeNP. The expression of genes encoding caspase-3 and caspase-4 was also increased by 2.6 and 2.9 times, respectively. This increase in expression may occur in response to the release of cytochrome C into the cytoplasm as a result of activation of the mitochondrial pathway. Incubation of A-172 cells with 5 µg/mL SeNP also increased the expression of mitogen-activated kinase genes in MAP3K5 and MAPK-8 cells by 2.5 times.

When cells were treated with Gap26 virtually no significant changes were observed in gene expression for most of the studied pro-apoptotic proteins, except for CHOP, GADD34, and BAX by 1.7, 1.8, and 1.5 times, respectively (Figure 7E, black columns). Treatment of A-172 cells with a Gap26 for 30 min and subsequent 24 h incubation with 5µg/mL SeNP caused an increase in some pro-apoptotic genes, GADD34, BIM, PUMA, which turned out to be 1.5–2 times lower than data on their expression obtained after treatment of cells with only 5 µg/mL SeNP (Figure 7E, green bars). The level of CHOP gene expression was the same as after incubation with nanoparticles and increased more than three times compared to intact cells. These results require additional confirmation for their correct interpretation. The expression levels of the other genes studied in the work, key participants in various pathways of apoptosis, practically did not differ in these samples compared to the control (intact cells). Interestingly, the expression level of the Gja1 gene encoding Cx43 increased 4-fold under the action of 5µg/mL SeNP and was not suppressed by the Gap26 blocker, an increase in expression 3.2-fold compared to intact cells (Figure 7F). Similarly, the expression level of the P2Y12 gene encoding P2Y-purinoreceptors, a key receptor in the signaling of SeNP, increased 3.2 times after incubation with SeNP and 2.6 times against the background of Gap26+SeNPs. At the same time, there was no significant change in the expression level of the Panx1 gene encoding pannexins, which are not involved in the signaling of SeNP (Figure 7F).

## 4. Discussion

Recently, more and more works have appeared on the study of the anticancer effect of selenium, selenoproteins, and SeNP [32,33,34]. The anticancerous property of SeNP is due to the induction of glutathione S-transferase (GST) by selenium [35]. SeNP have the potential to suppress the growth of cancer cells via the induction of cell cycle arrest at S phase [36]. Cancer cells selectively incorporate SeNP via endocytosis and then SeNP induces the apoptosis of cancer cell by triggering apoptotic signal transduction pathways [37]. SeNP in MCF–7 cancer cells cause an increase in the expression of cytochrome C, Bax, and P—p38, a decrease in CD44, which leads to disorganization and dysregulation of intracellular cytoskeleton F-actin, and induction of apoptosis and necrosis [38,39]. SeNP have been observed to inhibit the growth of prostate cancer cells (LNCaP) moderately via caspases mediated apoptosis in vitro [40]. At the same time, no connection was shown for the anticancer action of SeNP through the regulation of Ca^2+^ homeostasis. At present, the high-throughput screening method [41] is actively used to search for effective methods of anticancer therapy and in our further studies it can be used to search for intracellular SeNP targets.

An increase in [Ca^2+^]_i_, which occurs due to the mobilization of Ca^2+^ ions from the ER, is one of the key triggers of the initial stage of apoptosis. It has been shown that under the action of the Ca^2+^-ATPase ER blocker, Thapsigargin, cyt c is released [42]. This may be one of the mechanisms of apoptosis induction under the action of SeNP, which leads to the activation of IP_3_R and an increase in [Ca^2+^]_i_. SeNP is often used as carriers of chemotherapeutic agents such as cisplatin, 5-fluorouracil, doxorubicin, and irinotecan [43,44,45]. It is known that cisplatin is used in treatment of solid tumors and modulates the release of [Ca^2+^]_i_ from ER. In neuroblastoma and HeLa cells, cisplatin modulates the expression of IP_3_R and RyR, activating apoptosis [46,47]. An increase in [Ca^2+^]_i_ leads to the activation of a number of kinases responsible for the induction of apoptosis. At the same time, mitochondrial overload with Ca^2+^ ions and the opening of mitochondrial permeability transition pores (PTP) can occur, also causing apoptosis [48]. Apoptosis can be activated due to the mobilization of Ca^2+^ from the ER and its entry into mitochondria at the sites of direct contact with ER [49,50]. At the same time, a close spatial relationship between mitochondria and IP_3_R ER is known [51]. Of great interest is the study of Ca^2+^ dynamics in cancer cells using 3D models of glioblastoma, which makes it possible to take into account the cell–cell and cell–environment interactions [52].

Interestingly, a series of repetitive Ca^2+^ spikes in HeLa cells caused by UV or TNFα, occurring due to the mobilization of Ca^2+^ ions from the ER, precede the release of cyt c, and suppression of these increases in [Ca^2+^]_i_ by BAPTA or heparin inhibits the release of cyt c from mitochondria and induction of apoptosis [53]. Since in our experiments SeNP induce both Ca^2+^ transients and Ca^2+^ oscillations, which is then accompanied by the induction of an early stage of apoptosis, it can be assumed that this mechanism is activated as well. It is known that Bcl-2 family proteins play a major role in controlling the permeabilization of the mitochondrial outer membrane during apoptosis. At the same time, it was shown that some of the Bcl-2 family proteins could interact with the IP_3_R at the ER [54]. BAX through Ca^2+^-dependent interaction with mitochondria and ER leads to the induction of apoptosis [55].

It was found that in glioblastoma cells and malignant mesothelioma cells expressing Cx43, a reduced level of expression of antiapoptotic Bcl-2 and Src was observed which promoted carcinogenesis [15,56]. Suppression of the expression of Cx43 using anti-sense cDNA transfection [57] or by treatment of cells with antisense oligonucleotides [58] led to a violation of the regulation of cell growth. Fibroblast proliferation from cx43-knockout mice was significantly increased compared to cells expressing Cx43 [59]. Pharmacological inhibition of gap junctions suppressed apoptosis induced by streptonigrin (intrinsic apoptotic pathway) or to a-Fas (extrinsic apoptotic pathway) [60]. Blockade of intercellular communication via gap junctions attenuated the extent of apoptosis, for example, in BC31 cells [61], granulosa cells [62], or astrocytes [63]. At the same time, the activation of gap junctions in response to the application of SeNP, including the increased expression of the gene encoding Cx43, led to the activation of apoptosis and the anticancer effect in our experiments.

In this work, in response to the application of SeNP to A-172 cells, activation of Cx43, ATP secretion, activation of purinergic receptors, and the spread of Ca^2+^ signals throughout the cells were observed. This ultimately led to the induction of apoptosis in these cells.

These results are in good agreement with the studies by Zhang et al., which showed that, in breast cancer cells and glioma cells, tamoxifen increased [Ca^2+^]_i_ in the form of Ca^2+^ oscillations and waves by activating purinergic signaling, which contributed to increased cytotoxicity in cancer cells [64].

In addition, we have shown an increase in the expression of the pro-apoptotic genes CHOP, GADD34 BIM, PUMA, and BAX, which confirms the activation of apoptosis, most likely through the mitochondrial pathway. An increase in the level of expression of caspase-4 may indicate the activation of the internal ER-mediated pathway of apoptosis, as was previously established [65,66,67,68]. In this study, 5 µg/mL SeNP also promoted an increase in the expression of mRNA of mitogen-activated kinases in MAP3K5 and MAPK-8 cells, which suggests the activation of an alternative mitogen-activated kinase pathway for apoptosis [69].

It is known that CHOP is a multifunctional transcription factor and plays an important role in regulating the response to a wide range of cellular stresses, causing cell cycle arrest and apoptosis in response to ER-stress [70]. The expression level of the gene encoding this factor increased both in response to the Cx43 blocker and upon application of SeNP, including in the presence of Gap26. This indicates the pro-apoptotic action of SeNP, including through an alternative signaling cascade.

## 5. Conclusions

SeNP enter human glioblastoma cells through clathrin-associated endocytosis and pinocytosis and, through the activation of connexin-43, induce vesicular ATP secretion and activation of purinergic receptors of neighboring cells. The secreted ATP through the phosphoinositide signaling cascade leads to the mobilization of Ca^2+^ ions from the Thapsigargin-sensitive ER pool and activates the paracrine cell activation mechanism, which induces apoptosis at the physiological level. Inhibition of the mechanisms of apoptosis caused by SeNP occurred by blocking Cx43 with a peptide mimetic. Thus, a new mechanism of anticancer action of SeNP, involving the activation of the Ca^2+^ signaling system and paracrine regulation by activating the gap junction through the activation of connexin43, has been shown.

## Figures and Tables

**Table 1:** Primer sequences for real-time polymerase chain reaction (RT-PCR).

Gapdh	Forward 5′–ACATCGCTCAGACACCATG Reverse 5′–GCCAGTGAGCTTCCCGTT
Chop	Forward 5′–GCTCTGATTGACCGAATGG Reverse 5′–TCTGGGAAAGGTGGGTAGTG
Gadd34	Forward 5′–GACGAGCGGGAAGGTGTGG Reverse 5′–CTCCGAGAAGGTCACTGTCC
Bim	Forward 5′–GGACGACCTCAACGCACAGTACGAG Reverse 5′–GTAAGGGCAGGAGTCCCA
Puma	Forward 5′–CCATTCGTGGGTGGTCTTC Reverse 5′–CAGATATGCGCCCAGAGAT
Cas–3	Forward 5′–GCATTGAGACAGACAGTGGTG Reverse 5′–AATAGAGTTCTTTTGTGAGCATG
Cas–4	Forward 5′–CACGCCTGGCTCTCATCATA Reverse 5′–TAGCAAATGCCCTCAGCG
Bax	Forward 5′–GGGCTGGACATTGGACTTC Reverse 5′–AACACAGTCCAAGGCAGCTG
Bak	Forward 5′–GAGAGTGGCATCAATTGGGG Reverse 5′–CAGCCACCCCTCTGTGCAATCCA
Map3k5	Forward 5′–AACACCTGAAGCTTAAGTCCC Reverse 5′–TCAATGATAGCCTTCCACAGTG
Mapk–8	Forward 5′–AAAGGGAACACACAATAGAAGAG Reverse 5′–GCTGCTGCTTCTAGACTG
Panx1	Forward 5′–ACGCTGTTTGTTCCATTCCGAC Reverse 5′–CTCCATTATTTGCTTTAGTTTCAC
Gja1	Forward 5′–CCTATGTCTCCTCCTGGGTAC′ Reverse 5′–TCTGCTTGAAGGTCGCTGGTC′
P2Y12	Forward 5′–CGAGGGGTGTAGGTAAAGTCC Reverse 5′–GGGGCACTTCAGCATACTTATC′

## Data Availability

Data will be made available on reasonable request.

## Supplementary Materials

The following are available online at https://www.mdpi.com/article/10.3390/biology10080743/s1, Figure S1: The SeNP concentrations used in experiments to measure the dynamics of [Ca^2+^]_i_, 2.5 µg/mL (A) and 5 µg/mL (B) after 30 min of incubation do not cause apoptosis and necrosis in A-172 cells.
