# Supplementary material for: Mechanism of Ca2+-Dependent Pro-Apoptotic Action of Selenium Nanoparticles, Mediated by Activation of Cx43 Hemichannels
Source: Biology (Basel). 2021 Aug 3;10(8):743. doi: 10.3390/biology10080743 (PMC8389560; doi:10.3390/biology10080743)
Supplement: Supplementary file 1 [file biology-10-00743-s001.zip › biology-1297406-supplementary.pdf]

biology MDPI

Supplementary

Mechanism of Ca^{2+}-Dependent Pro-Apoptotic Action of Selenium Nanoparticles, Mediated by Activation of Cx43 Hemichannels

Egor A. Turovsky and Elena G. Varlamova

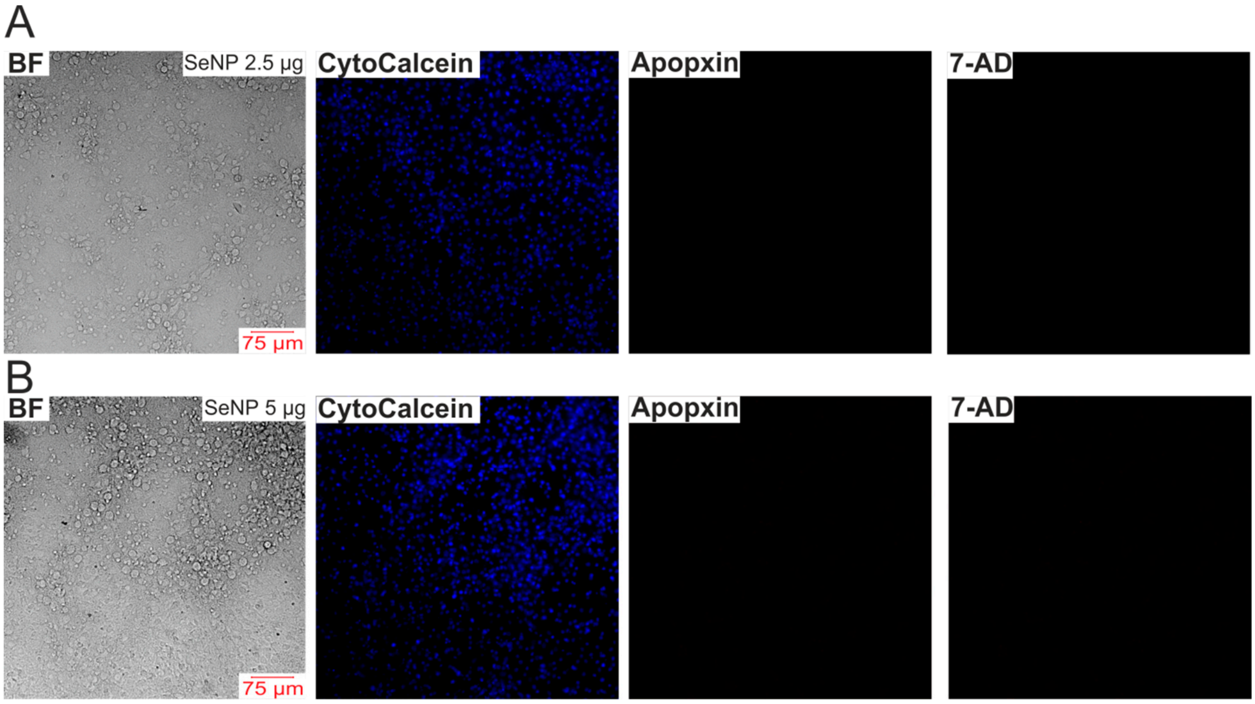

Figure S1. The SeNP concentrations used in experiments to measure the dynamics of $[Ca^{2+}]_i$, 2.5 µg/ml (A) and 5 µg/ml (B) after 30 minutes of incubation do not cause apoptosis and necrosis in A-172 cells. BF – bright-field microscopy, CytoCalcein – living cells indicator, Apopxin – apoptotic cells indicator and 7-AD (7-aminoactinomycin D) – necrotic cells indicator.
